# What Do These Findings Tell Us? Comment on Tinella et al. Cognitive Efficiency and Fitness-to-Drive along the Lifespan: The Mediation Effect of Visuospatial Transformations. *Brain Sci.* 2021, *11*, 1028

**DOI:** 10.3390/brainsci12020165

**Published:** 2022-01-27

**Authors:** Robert E. Kelly, Anthony O. Ahmed, Matthew J. Hoptman

**Affiliations:** 1 Department of Psychiatry, Weill Cornell Medicine, White Plains, NY 10605, USA; aoa9001@med.cornell.edu; 2 Clinical Research Division, Nathan S. Kline Institute for Psychiatric Research, Orangeburg, NY 10962, USA; matthew.hoptman@nki.rfmh.org or; 3 Department of Psychiatry, New York University Grossman School of Medicine, New York, NY 10016, USA

Tinella et al.’s recent article [1] seems like a natural extension of their previous work [2], wherein the authors provided evidence to “suggest the specific contribution of spatial mental transformation skills in the execution of complex behaviors connected to the fitness to drive”. Their more recent paper extends this work by performing a path analysis to explore mediating pathways that can, in part, explain some of the variance in the prediction of driving skill scores. However, additional information would facilitate our understanding of this paper’s contribution to science, specifically by addressing the questions “Do these results seem replicable?” and “How do these results advance our understanding of brain function and/or human behavior?” The purpose of this comment is to call attention to these important questions, which often remain unanswered in published scientific papers, and to provide an opportunity for the authors to answer these questions more fully.

In recent years, growing concerns have been raised in the scientific community about the high rates of non-replication of research results across a wide variety of scientific endeavors—much higher than the conventional α = 0.05 used for the statistical testing of research hypotheses [3,4,5,6,7,8,9]. These high rates of non-replication may, in part, be expected from Bayesian statistics [10], but other major contributing factors include methodological errors [11], poorly powered studies [8,12], and the reporting bias that results from academic pressures to publish in combination with a publication bias favoring positive, “statistically significant” findings [9,13]. This reporting bias inflates effective *p*-values because no correction is made for unreported alternative hypotheses that were tested. The problem is further exacerbated by practices such as hypothesizing after the results are known (HARKing) [14], adjusting parameters of the data-processing pipeline and statistical analyses until the sought-after, statistically significant results are “found”, also known as *p*-hacking [10,15,16,17,18], and other related practices [19]. Even the data analyses for uncomplicated studies can thus be manipulated, for example, by adjusting the inclusion/exclusion criteria for participants in a study. To minimize these effects, authors should include sufficient methodological detail in their papers to allow readers to properly evaluate the relevance of the chosen methods [17,18].

For example, the two articles by Tinella et al. lack important details concerning the selection of study participants, leading to puzzling discrepancies in the participant selection criteria for the two studies, which were by the same authors, studying the same variables, in the same location. The earlier study included 120 males and 63 females, ages 18–64, whereas the more recent study included only 117 males, ages 18–64, and added 58 males, ages 65–91. So, why were 3 of the males and all 63 females excluded for the recent study? Why did the authors choose to add males only in the 65–91 age group? Why would these studies’ inclusion/exclusion criteria differ at all? Data collection is costly, so it would be of interest for readers to know what prompted the authors to discard much of the data for the recent study.

Moreover, regression-based analytic methods such as mediational models, path analysis, factor analysis, and structural equation models potentially capitalize on chance. The regression weights or path coefficients that depict the magnitude of associations of arrows in path diagrams are maximized for the study sample, in keeping with the least-squares criterion. This is a long-recognized challenge in regression and a source of criticism for path-analytic models [20,21]. A common solution is to designate and fit the original hypothesized model in “training” data and then attempt a replication in an independent cross-validation dataset [22,23]. The degree of replicability could then be inferred by examining the precision efficacy, proportional shrinkage, and/or prior predictive *p*-values [24].

The meaning of the derived study findings also merits explanation. For example, how does the finding that “mental rotation” (MRT) partially “mediates” the relationship between “global cognitive functioning” (Montreal Cognitive Assessment, MoCA) and “resilience of attention” (Determination Test, DT) contribute to our understanding of brain function or human behavior? This finding itself is not surprising: the MoCA includes some visuospatial testing and all of the studied variables correlate with each other to some degree. Sometimes there is a value in confirming expectations, but, in this case, what does the finding tell us in terms of latent constructs reflecting brain function? In particular, what is meant by the construct “global cognitive functioning”, which the authors describe as corresponding to the observable MoCA scores? The MoCA was developed as a measure of cognitive impairment in elderly persons suspected of developing dementia or mild cognitive impairment and was validated on a sample of three groups of elderly adults: Alzheimer’s disease, mild cognitive impairment, or no cognitive impairment [25]. Although the MoCA serves well to predict cognitive impairment and to identify persons having Alzheimer’s dementia or mild cognitive impairment [26], its meaning becomes unclear when applied to a sample of cognitively intact individuals.

To illustrate the point, consider the following analogy. Suppose we were to use student height to predict student age among students in secondary school, grades 1–12. If we were to limit our study to include equal numbers of students from grades 1, 5, and 12, we would surely find that height is an excellent predictor of age and, further, that height is also an excellent predictor of age for virtually any large sample of secondary school students randomly drawn from grades 1–12. However, height would be a terrible predictor of age for samples randomly drawn from grade 12 only (Figure 1). In the same way, we should not anticipate that MoCA score will reflect “cognitive impairment” in healthy controls from ages 18–91 as well as it did in studies validating the MoCA as a useful measure of cognitive impairment. Compared with cognitively impaired people, we can expect that differences in MoCA scores among healthy controls would reflect confounding variables in the prediction of cognitive impairment, such as education level [27] and intelligence [28], to a greater degree. Many other variables could be considered, such as “physical exercise” or “mental exercise”. It is not clear what is being measured when the MoCA is applied to healthy adults, so what does MoCA score tell us about brain function in the current study?

Finally, the question concerning what this study tells us about human behavior also merits consideration. The MoCA is a natural subject of study, given its use in real-world applications concerning fitness-to-drive among individuals thought to be cognitively impaired due to dementia. Having moderate to severe dementia is considered evidence of unfitness-to-drive [29], so the MoCA score, weighed together with other relevant clinical information, can help to determine the diagnosis of dementia and fitness to drive. However, the relevant question here is “What does a study of cognitively intact men tell us about fitness to drive among cognitively impaired elderly adults?” Why not directly study cognitively impaired adults?

In summary, important questions remain unanswered in the article in question, concerning participant inclusion/exclusion criteria, replicability of the path analysis, and implications concerning brain function and human behavior. Elucidating these issues might better enable readers to evaluate this study’s contribution to advancing scientific knowledge.

## Figures and Tables

**Figure 1:**
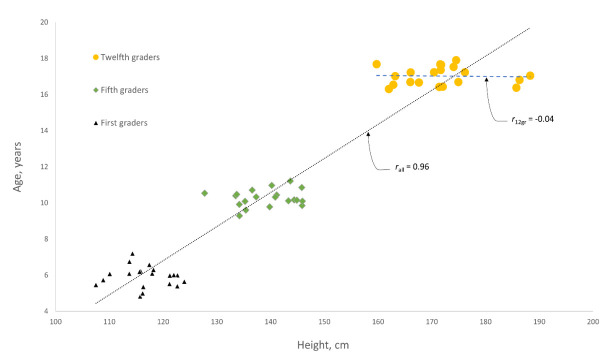
Scatterplot of student age vs. height, showing that height is an excellent predictor of age, by Pearson’s *r,* for all students combined (*r*_all_), but not for students in grade 12 only (*r*_12gr_). This illustration depicts secondary school students from grades 1, 5, and 12 using simulated data. The dotted and dashed lines show the lines of best fit, using least squares linear regression, for all students and for students in grade 12 only, respectively.

## Data Availability

Data for the figure were generated using LibreOffice Calc version 5 for Linux to derive random pairs of values for height and age, based on the normal distribution. For first-graders, the mean and standard deviation for height (cm) were [115, 4], and for age (years) [6, 0.5]. The corresponding values for fifth-graders were height [139, 5] and age [10, 0.5]. For twelfth-graders, they were height [170,9] and age [17, 0.5].
